# Genome Wide Identification and Characterization of *BrE2F* Family Gene of *Brassica rapa*

**DOI:** 10.1155/ijog/7106391

**Published:** 2026-06-15

**Authors:** Naima Sultana, Md. Jahid Hasan Jone, Anjan Chandra Sharma, Setu Rani Saha, Mohammad Rashed Hossain, Shirin Akhter, Md. Anisur Rahman, Jobadatun Naher

**Affiliations:** ^1^ Genetics and Plant Breeding, Bangladesh Agricultural University, Mymensingh, 2202, Bangladesh, bau.edu.bd; ^2^ Department of Agricultural Extension, Ministry of Agriculture, Mymensingh, Bangladesh, agriculture.tn

**Keywords:** *Brassica rapa*, E2F gene, genome, miRNA, protein–protein interaction, stresses

## Abstract

The *E2F* family gene is one of the most important G_1_–S checkpoint regulatory transcription factors to monitor and regulate the accurate DNA replication and progression of the cell cycle in eukaryotes. Expression of this gene in multiple environmental factors has been reported in diversified plant species. However, characterization and investigation of the putative influential role of this family gene in *Brassica* species were not reported. In addition to expression profiling and mRNA target analysis, we have comprehensively identified and characterized 14 *Br*E2F/DP transcription factors from the data in available databases of *Brassica rapa* and compared them with five other species. Six pairs of segmentally duplicated *BrE2F/DP* paralogous genes were identified in this study, with Ka/Ks values of < 1.0. The findings indicated their evolution through segmental duplication across the chromosome after the *Brassica* splits from *Arabidopsis* and functional similarities with *Brassica oleracea* proteins. Most of the stress‐responsive microRNA (miRNA) targeted *BrE2F/DP5*, *BrE2F/DP12*, BrE2F/DP1, and BrE2F/DP8. Cis‐element composition and diversified functional expressions indicated their involvement in biological pathways for adaptation under multiple stress resistances, including cell cycle progression during growth and development. The findings provided comprehensive information on the multifunctional roles of the E2F/DP family in *Brassica* spp., importantly, biotic and abiotic stress resistance.

## 1. Introduction

The E2F is a family of transcription factors playing a crucial role in cell cycle regulation. They bind to promoter regions of multiple target genes for controlling cell proliferation either by suppression or by activation of their expression [1, 2]. With increasing the structural and functional investigation of this transcription factor, it has become evident that this family regulates hundreds of potential genes involved in DNA replication at S‐phase, including many other noncell cycle regulatory genes such as those for apoptosis, signal transduction, cellular biogenesis and protein destination, DNA repair, and stress response both in plants and animals [3, 4]. However, around 31.3% of its target genes are for cell cycle regulation and DNA replication [5]. Since it was first introduced in 1987 [6], the consecutive study identified its eight member proteins (from animals) with distinctive roles and functionally grouped them into (1) activators (E2F1‐3: induce cell division and endoreplication by activating gene expression) and (2) repressors (E2F4‐8: inhibit cell division by repressing gene expression) [1, 7]. Structurally, the E2F family is further divided into two members based on their interaction with DP proteins: (1) “typical”: subfamily E2F1 to E2F6, having one DNA‐binding domain adjacent to a dimerization domain to interact with DP proteins (DP1 and DP2) to form a heterodimer complex and (2) “atypical”: subfamily E2F7 and E2F8 contain two distinct DNA‐binding subdomains and control the expression of target genes independent of DPs [8]. Studies have indicated that atypical E2Fs originated either via duplication of classical E2F genes or by the substitution of the DP‐binding domains of typical E2Fs by a second DBD [8]. However, the evolution of E2Fs has still remained unclear. Therefore, in this study, we designated the E2F gene members as E2F/DP. Nevertheless, the number of genes varied in species. In *Arabidopsis thaliana,* among six *AtE2F*‐identified genes, *AtE2Fa*, *AtE2Fb*, and *AtE2Fc* belong to “typical” “activator” (conserved domains have similarities with other plant and animal E2Fs), and *AtE2Fd*/*DEL2*, *AtE2Fe/DEL1*, and *AtE2Ff/DEL2* belong to “atypical” “repressor” or *DEL (DP-E2F*‐*like*) [9, 10]. In the rice genome, four E2Fs, three DPs, and two DELs [11] and in the *Medicago truncatula* genome, three E2Fs and one DP and DEL were identified [12]. However, in *Brassica napus* this gene family was classified into *E2Fs* (*E2FA to E2FF*) and *DPs* (*DPA* and *DPB*) [13].

Due to a great extent of protein sequence homology (around 22%) in the DNA‐binding regions, E2F forms a large heterodimeric complex with a dimerization partner (DP) [8, 14]. Their inactivation by retinoblastoma (RB/E2F/DP) and activation by CDK/cyclin complexes are the central players in expressing the genes for transition from G1 to S‐phase [15]. In plants, this complex binds to the TTTCCCGCC motif of the promoter region, mostly within 400 bp upstream from the putative ATG initiator codons of its target genes [5]. In *Arabidopsis* three typical E2Fs bind with two types of DPs (DPa and DPb). In addition to a common DNA‐binding domain and dimerization domain, E2Fs contain a “marked box” domain and Rb‐binding domain [14]. The E2Fs and DPs dimerize with conserved dimerization domains to interact with the canonical E2F motif to regulate the cell cycle. However, co‐expression of *AtE2Fa* and *AtE2Fb* with *DPa* regulates cell division positively, whereas *AtE2Fc* with *DPb* regulates it negatively [16].

Due to the disturbance of the cell cycle by biotic and abiotic stress, plant growth is modulated either for adaptation or to come to the end of its life cycle [12]. Some *E2F/DP* genes show higher expression in the cells at the quiescent phase (G0), indicating potential roles outside the cell cycle, particularly in stress adaptation [17]. *E2F* genes are differentially expressed in environmental stress; even the same gene varies in its way of expression at different plant organs under the same stressor [18]. Many stress response genes were also reported to be regulated by these genes to facilitate better growth [18, 19]. The important role of the expression of these genes in stress defense was reported in wheat, tomato, mustard and rice [13, 18, 20]. For example, in *B. napus* co‐expression of *E2Fs* and *DPs* genes increased under diverse abiotic stresses [13]. Under drought stress, water use efficiency was increased in *Arabidopsis* by the E2Fa‐dependent EDT1/HDG11‐ERECTA genetic pathway through increasing leaf cell size and reducing stomatal conductivity by reducing the rate of cell division and promoting endoreduplication [21].

However, comprehensive genome‐wide analyses of this gene family have been limited to a few plant species. *Brassica* crops are worldwide cultivated due to their importance in industrial as well as food for both human consumption (as oil and vegetables) and animal fodder. Despite their importance, the evolutionary relationships and functional characteristics of these family genes within Brassicaceae remain largely unexplored, because, until now, the study of this gene was limited only to *B. napus* [13]. Among the species of Brassicaceae, *Brassica rapa* has potential for use as a model species due to the identification of its complete genome sequence and proteins (nearly 40,000) [22] and its phylogenic relationship to other model species (*Arabidopsis* *thaliana*) in addition to significant economic importance.

Hence, in this study, we have comprehensively investigated E2F genes in the *B. rapa* genome. Thereafter, gene structure, conserved motifs and domains, multiple sequence alignment, its phylogenetic and evolutionary relationship with other species, duplication, protein structure, and protein–protein interaction (PPI) networks were analyzed. Additionally, to understand their functional role in *B. rapa*, cis‐acting elements, microRNA (miRNA) target sites, and RNA sequencing data were analyzed. This study serves as a reference for future research on the structure and function of the E2F/DP transcription factor in *B. rapa*. In addition to some knowledge on evolutionary ancestors, their involvement in multiple biological pathways related to growth, development, and stress resistance has been elucidated.

## 2. Materials and Methods

### 2.1. Identification, Sequence Retrieval, and Validation of BrE2F/DP Family Genes

To investigate the *E2F/DP* gene family at the genome‐wide level in *B. rapa*, we retrieved 14 *BrE2F/DP* gene sequences from the *Brassica* Database (BRAD) (https://brassicadb.cn). To confirm the identities of the retrieved genes, we conducted BLASTp analyses against the NCBI database using *A. thaliana E2F/DP* protein sequences as queries. For further validation, the hidden Markov model (HMM) profile of the DNA‐binding domain (PF02319) was obtained from the Pfam database (https://pfam.xfam.org) and employed to accurately identify E2F/DP members. We then collected coding DNA sequences (CDS), protein sequences, and genomic data of the BrE2F/DP genes (as listed in the Supporting Data File) from the BRAD database. The authenticity of these sequences was subsequently confirmed in the iTAK database (http://itak.feilab.net/cgi-bin/itak/index.cgi). The physicochemical parameters of the BrE2F/DP proteins were analyzed using the ProtParam tool (https://www.expasy.orgprotparam). Meanwhile, the Ensembl Plants database (https://plants.ensembl.org) was employed to identify open reading frames (ORFs) of the BrE2F/DP genes. Protein localization within subcellular compartments was derived through the Cell‐PLoc 2.0 web service package. The protein sequences of these genes were retrieved in TAIR (https://www.arabidopsis.org) and iTAK for *A. thaliana* (7), *Brassica oleracea* (19), *Oryza sativa* subsp. *japonica* (9), and *Solanum lycopersicum* (8). Each protein sequence of all the species was further subjected to analysis using the HMMER web server for verification of the presence of their DNA‐binding domain.

### 2.2. Conserved Sequence Analysis and Phylogeny of BrE2F/DP Proteins

The complete E2F/DP protein sequences of *B. rapa*, *A. thaliana*, *B. oleracea*, *O. sativa* subsp. *japonica*, and *S. lycopersicum* were aligned by default parameters and the ClustalW algorithm in DNAMAN (Version 10.0) (https://www.lynnon.com/dnaman.html) and the alignment results were visualized with GeneDoc (https://www.nrbcs.orggfxgenedocebinet.htm). Phylogenetic analysis was carried out in MEGA (Version 11.0.13) employing the neighbor‐joining (NJ) method [23] (parameters: Pearson correction, pairwise deletion, and bootstrap values in percentages with 1000 replicates) to assess the evolutionary connections among the E2F/DP proteins.

### 2.3. Analysis of Conserved Motifs, Gene Structure, and Domain Prediction

The MEME Suite v5.1.0 tool (http://meme-suite.org/tools/meme) was used to identify and characterize conserved motifs within BrE2F/DP proteins. To detect both short and extended conserved regions, the analysis parameters were configured to identify up to ten distinct motifs within the protein sequences, with a minimum width of six and a maximum width of 50 amino acids. By utilizing the GSDS web tool (https://gsds.gao-lab.org/), gene structures were depicted comprising exons, introns, and flanking sequences. To analyze exon and intron distribution, genomic sequences were aligned with the corresponding coding sequences (CDS) of the *BrE2F/DP* genes. Conserved protein domains for the 14 *BrE2F/DP* genes were predicted through two online platforms: the SMART database (http://smart.emblheidelberg.de/) and the NCBI Conserved Domain Database (CDD) tool (https://www.ncbi.nlm.nih.gov/Structure/bwrpsb/bwrpsb.cgi).

### 2.4. Chromosomal Locations and Synteny Analysis

The chromosomal coordinates, including start and end positions, chromosome number, and gene length, for the 14 BrE2FDP genes were retrieved from the Ensembl Plants database. The fourteen *BrE2F/DP* genes in the 10 chromosomes of the *B. rapa* genome were mapped using the MapGene2 Chromosome v2 web tool (http://mg2c.iask.in/mg2c_v2.0/) based on their physical location. Genome annotation files (.gff) and sequence data (.fa) for *A. thaliana* were obtained from the TAIR database, while the corresponding files for *B. oleracea*, *O. sativa*, and *S. lycopersicum* were acquired through the Phytozome platform. To investigate conserved gene relationships, synteny analysis between *B. rapa* and the other species was carried out using the MCScanX software. Visualization of collinear gene blocks was performed with TBtools.

### 2.5. Gene Duplication Analysis and Calculation of Ration of Nonsynonymous and Synonymous (Ka/Ks)

Potential duplicated BrE2FDP genes were identified using an NCBI BLAST search based on high sequence similarity and coverage between the 14 BrE2FDP genes. These duplicated pairs were then used to estimate the extent and timing of duplication events by calculating nonsynonymous rate (Ka), synonymous rate (Ks), and the Ka/Ks ratio, which indicates the type of selection pressure acting on the genes [24]. Initially, protein sequences of the gene pairs were aligned using Clustal Omega. The resulting alignments, along with their cDNAs, were converted into codon‐based alignments using the PAL2NAL web tool. These codon alignments served as input for the CODEML program in the PAML suite to calculate the Ka and Ks values. Divergence times were estimated using the formula T = Ks/(2*λ*), where the Ks value reflects the rate of neutral mutations, is used as an evolutionary time, and *λ* represents the rate of synonymous substitutions (assumed to be 1.5 × 10^−8^ per site per year). Finally, the Ka/Ks ratio was used to infer whether the gene pairs were evolving under purifying, neutral, or positive selection.

### 2.6. Identification of Cis‐Regulatory Elements and Functional Analysis of BrE2FDP Genes

Using the PlantCARE database, regulatory cis‐elements (around 5–10 bp in length) were analyzed in 14 BrE2FDP genes. Meanwhile, the Blast2GO functional annotation and genomics software provided insights into the molecular functions, biological roles, and subcellular distribution of their corresponding proteins.

### 2.7. Expression Profiling of BrE2F/DP Genes

To study *BrE2F/DP* gene expression, RNA‐seq data were obtained from the Expression Atlas (https://www.ebi.ac.uk/gxa/home). The dataset included six types of tissue from *B. rapa*, including leaf, stem, flower, silique, root, and callus. Expression levels, originally measured in FPKM (fragments per kilobase of exon per million mapped reads), were normalized across genes usingZ‐scores. A heatmap visualizing gene expression was then constructed using the Complex Heatmap package in *R*.

### 2.8. Prediction of miRNA Target Sites

A Plant Small RNA Target Analysis Server (https://www.zhaolab.orgpsRNATarget) was used to explore potential miRNAs targeting *BrE2F* genes. Genomic sequences of all *BrE2F* genes were submitted to the psRNATarget web tool against the reference genome (*B. rapa*) with the default settings. Cytoscape software was later used to present the regulatory mechanism of miRNAs and their target *BrE2F* genes.

### 2.9. Protein‐Protein Interaction, Protein Homology Modeling and Sequence Similarity

The String Database tool was used to predict and visualize protein–protein association networks of BrE2F/DP proteins. Protein homology modeling was performed with the help of the Swiss Model [25] to create predicted three‐dimensional structures through template recognition of known homologous protein structures. Furthermore, TBtools was utilized for performing sequence similarity analysis to assist in comparing amino acid sequences and constructing a similarity matrix.

## 3. Results

### 3.1. Identification of E2F/DP Family Genes and Sequence Retrieval

Fourteen *E2F/DP* genes were identified in the *B. rapa* genome, which were designated chronologically as *BrE2F/DP1* to *BrE2F/DP14* and listed in Table 1 accordingly. Their corresponding proteins were retrieved and classified into three functional groups: (i) E2F; (ii) DP; and (iii) DEL through the BLASTp search of *Arabidopsis E2F/DP* genes. Six of these proteins belonged to the E2F group, whereas each of the DP and DEL groups has 4 proteins. There was considerable variation in CDS length, ranging from 852 bp (*BrE2F/DP3*) to 2862 bp (*BrE2F/DP6*), with their protein lengths from 283aa (32.35 kDa) to 953aa (105.92 kDa), respectively. The theoretical pI values of all the proteins were below 7. The GRAVY values of the *BrE2F/DP*s varied from −0.462 (*BrE2F/DP*12) to −0.801 (*BrE2F/DP*4). All the proteins were observed to be localized within the nuclear region.

**TABLE 1:** Detailed information on the *E2F/DP* genes and corresponding proteins in *Brassica rapa.*

Gene name	Gene ID	Group name	ORF (bp)	Chromosome location			Protein length (aa)	Mol. Wt. (KDa)	pI	GRAVY	Exons	Introns	Subcellular localization
				Number	Start	End							
BrE2F/DP1	Bra002398	E2F	1332	A10	9,903,517	9,906,922	443	49.07	4.79	−0.733	14	13	Nucleus
BrE2F/DP2	Bra005305	E2F	1446	A05	4,683,596	4,686,521	481	52.21	4.82	−0.526	13	12	Nucleus
BrE2F/DP3	Bra005726	DP	852	A03	307,028	308,707	283	32.35	7.72	−0.675	8	7	Nucleus
BrE2F/DP4	Bra005763	DP	1098	A03	476,707	478,631	365	40.35	5.77	−0.801	9	8	Nucleus
BrE2F/DP5	Bra006615	E2F	1392	A03	4,285,741	4,289,221	463	51.36	4.84	−0.766	14	13	Nucleus
BrE2F/DP6	Bra009530	DP	2862	A10	15,909,066	15,914,525	953	105.92	4.74	−0.747	20	19	Nucleus
BrE2F/DP7	Bra009586	DP	873	A10	16,135,525	16,137,297	290	32.91	7.70	−0.663	8	7	Nucleus
BrE2F/DP8	Bra014096	E2F	1104	A08	3,558,057	3,560,286	367	41.55	5.63	−0.680	13	12	Nucleus
BrE2F/DP9	Bra017268	E2F	1464	A04	15,756,017	15,759,327	487	52.95	4.91	−0.607	13	12	Nucleus
BrE2F/DP10	Bra018080	DEL	1155	A06	9,804,540	9,806,647	384	43.43	8.28	−0.708	11	10	Nucleus
BrE2F/DP11	Bra023047	E2F	1410	A03	8,405,920	8,408,905	469	51.35	4.80	−0.554	13	12	Nucleus
BrE2F/DP12	Bra023497	DEL	1299	A02	2,594,468	2,596,775	432	49.27	9.19	−0.462	10	9	Nucleus
BrE2F/DP13	Bra033767	DEL	1674	A01	13,892,387	13,896,483	557	62.66	6.60	−0.605	13	12	Nucleus
BrE2F/DP14	Bra039127	DEL	1077	A05	25,202,104	25,204,199	358	40.54	7.14	−0.704	11	10	Nucleus

*Note:* MWt: molecular weight, pI: iso‐electric point, GRAVY: grand average of hydropathy.

Abbreviation: ORF, open reading frame.

### 3.2. Multiple Sequence Alignment and Evolutionary Analysis of BrE2F/DP Proteins

**FIGURE 1:**
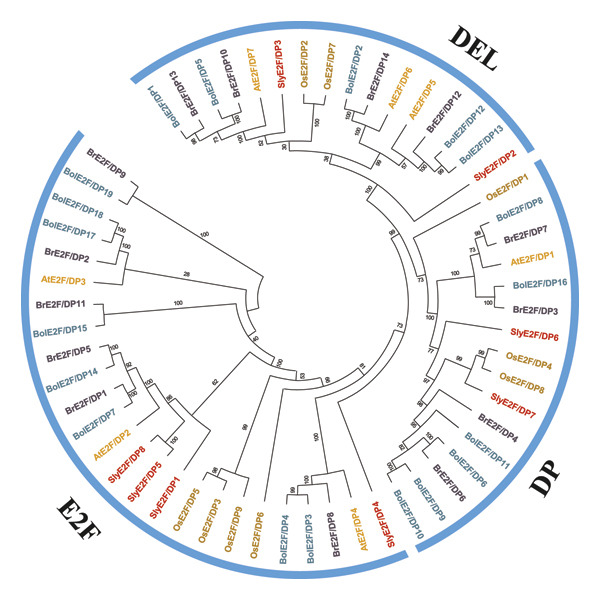
Phylogenetic tree of BrE2F/DP proteins. The tree was generated using MEGA software by neighbor joining method with 1000 bootstrap replicates. A species acronym was added before each E2F/DP protein name.

Multiple protein sequence alignment of *B. rapa* and four other plant species (*A. thaliana*, *B. oleracea*, *O. sativa*, and *S. lycopersicum*) within the E2F/DP family revealed the presence of three conserved domains: the DNA‐binding domain, the dimerization domain, and the Rb‐binding domain. These domains are detailed in Supporting File 1(SF1). Proteins in the E2F group possessed all three domains, whereas those in the DP group contained the DNA‐binding and dimerization domains, and proteins in the DEL group featured only the DNA‐binding domain. The phylogenetic analysis also clustered E2F/DP proteins into these three groups, with 25 members in E2F and 16 members in both DP and DEL (Figure 1). The groups were named according to *A. thaliana* E2F/DP proteins, and the E2F, DP, and DEL proteins of *B. rapa* clustered in these groups, respectively. Each group comprised clusters representing both monocot and dicot species. All BrE2F/DP proteins in the phylogenetic tree were represented by orthologous pairs (i.e.,BrE2F/DP11‐BolE2F/DP15, BrE2F/DP9‐BolE2F/DP19and BrE2F/DP5‐BolE2F/DP14). Furthermore, BrE2F/DPs exhibited the highest sequence similarity with BolE2F/DPs, followed by AtE2F/DPs, SlyE2F/DPs, and OsE2F/DPs in the phylogenetic analysis.

### 3.3. Conserved Motif Analysis, Domain Prediction and Exon–Intron Distribution

Variations in motif distribution were observed among different groups of BrE2F/DP proteins. Altogether, ten conserved motifs were identified within the BrE2F/DP family (Figure 2(a)), with Motif 1 being universally present across all 14 gene products. Proteins clustering within the same phylogenetic group (Figure 1) exhibited highly similar motif patterns. For instance, all E2F group members consistently contained motifs 1, 2, 3, 4, 5 and 8, whereas DP group members predominantly featured motifs 1, 5, and 7. Most DEL group proteins shared motifs 1, 3 and 6. These patterns suggest that closely related BrE2F/DP proteins tend to exhibit conserved structural features.

FIGURE 2Domain regions of BrE2F/DP proteins (a), ten conserved motifs (b) and exon‐intron distribution in the BrE2F/DP genes (c). A species acronym was added before each E2F/DP protein name. Different colored boxes represent different motifs and domain region. The blue boxes represent the exons and the red lines represent the introns.

**Figure:**
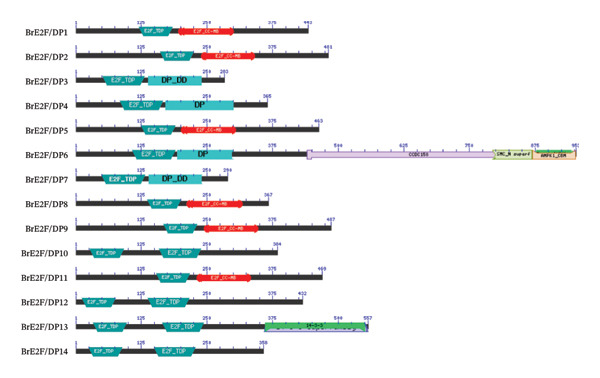
(a)

**Figure:**
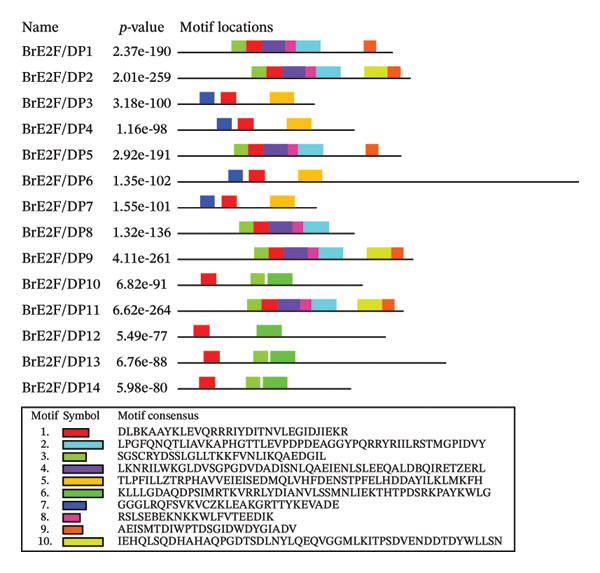
(b)

**Figure:**
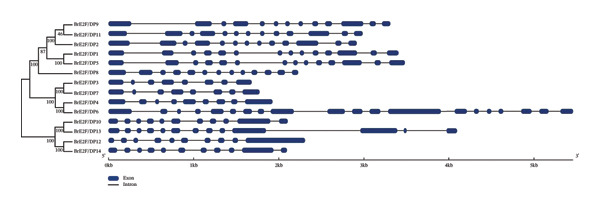
(c)

Domain architecture analysis further supported this structural conservation, revealing that BrE2F/DP proteins display varying domain compositions, ranging from the presence of the E2F_TDP domain as well as combinations with additional domains such as E2F_CC‐MB, DP and the DP_DD superfamily (Figure 2(b)). All BrE2F proteins harbored at least one E2F_TDP domain, indicating their capacity for DNA binding. The E2F_CC‐MB domain was found only in E2F group members, while the DP and DP_DD domains were restricted to the DP group. Interestingly, DEL group members possessed two E2F_TDP domains.

To further investigate the structural diversity of BrE2F/DP genes, exon–intron organization was analyzed (Table 1, Figure 2(c)) and the observation of intron numbers ranged from 7 to 19 (Table 1). Closely related genes within the same phylogenetic group displayed conserved exon–intron structures, both in number of introns and exon length (Supporting Figure 2), reinforcing the evolutionary conservation within groups. As an example, members of the E2F group such as *BrE2F/DP2*, *BrE2F/DP8*, *BrE2F/DP9*, and *BrE2F/DP11* each contained 12 introns. Similarly, *BrE2F/DP3* and *BrE2F/DP7* from the DP group possessed seven introns, while *BrE2F/DP10* and *BrE2F/DP14* from the DEL group had 10 introns each (Table 1). In contrast, genes such as *BrE2F/DP4*, *BrE2F/DP6*, *BrE2F/DP12*, and *BrE2F/DP13* exhibited variations in exon and intron numbers compared to other members within their respective groups, indicating possible structural divergence. In addition, except for *BrE2F/DP10* and *BrE2F/DP13,* all the duplicated gene pairs shared similar exon–intron distribution (Table 2, Figure 2(c)).

**TABLE 2:** Ka/Ks ratio and estimated divergence time for the duplicated *BrE2F/DP* paralogs.

Paralogous pairs	Query cover (%)	Identity (%)	Ks	Ka	Ka/ks	Date (Mya)	Duplication type
BrE2F1‐BrE2F5	100	80.60	0.2916	0.0697	0.2391	9.72	Segmental
BrE2F2‐BrE2F9	93	83.33	0.3290	0.1084	0.3296	10.97	Segmental
BrE2F2‐BrE2F11	93	82.86	0.3043	0.0931	0.3059	10.14	Segmental
BrE2F3‐BrE2F7	98	86.97	0.5708	0.0744	0.1304	19.02	Segmental
BrE2F9‐BrE2F11	92	82.06	0.3411	0.0893	0.2618	11.37	Segmental
BrE2F10‐BrE2F13	91	84.53	0.5345	0.1088	0.2035	17.82	Segmental

Abbreviation: Mya, million years ago.

### 3.4. Chromosomal Locations, Gene Duplication, and Synteny Analysis

Among 10 genomic chromosome pairs of *B. rapa*, *BrE2F/DP* genes were unevenly distributed on eight pairs (Figure 3(a)). The highest number (four) of these genes was located in chromosome A03, followed by chromosome A10 (three). Chromosome A05 contained two genes, while a single gene was found on each of chromosomes A01, A02, A04, A06, and A08. The sequence identity among the 14 BrE2F/DP proteins ranged from 28% (*BrE2F/DP1* and *BrE2F/DP12*) to 85% (*BrE2F/DP10* and *BrE2F/DP13*) (Figure 3(b)). Using criteria of over 80% query coverage and sequence identity (Supporting Figure 2), six pairs of segmentally duplicated *BrE2F/DP* paralogous genes were identified (Table 2). These gene pairs were separately located on A01, A03, A04, A05, A06, and A10 (Figure 3(a)). Consistent with phylogenetic analysis (Figure 1), all duplicated pairs were found within the same evolutionary group. In syntenic relations of *BrE2F/DP* genes with those of four other genomes, a total of 62 syntenic pairs were calculated (SF3, Figure 3(b)). The analysis revealed that *B. rapa* shared the highest number of syntenic blocks with *B. oleracea* (36), followed by *A. thaliana* (12) and *S. lycopersicum* (10). However, only 4 blocks were found with *O. Sativa*. The maximum number of syntenic blocks (9) was found in *BrE2F/DP8*, while no syntenic blocks were observed in *BrE2F/DP9* (SF3).

FIGURE 3Chromosomal distribution of *BrE2F* genes (a) and its syntenic relationships (b) with those of *Arabidopsis thaliana* (At), *Brassica rapa* (Br), *Brassica oleracea*, *Oryza sativa* (Os), and *Solanum lycopersicum* (Sly) and sequence identity (%). The length of the chromosomes is indicated by the scale on the left. The gray lines in the background represent the collinear blocks within *Brassica rapa* and other genomes, while the blue lines highlight the syntenic *E2F/DP* gene pairs. The red triangle corresponds to the gene location in the chromosome, Chr with number indicate chromosome number.

**Figure:**
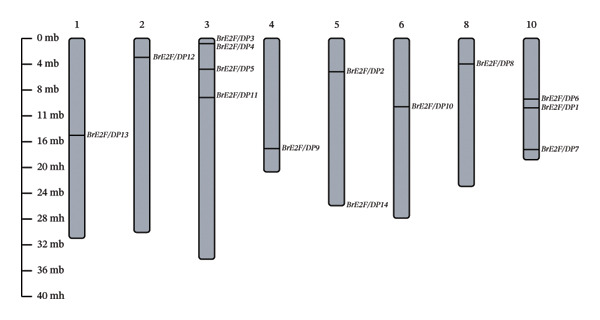
(a)

**Figure:**
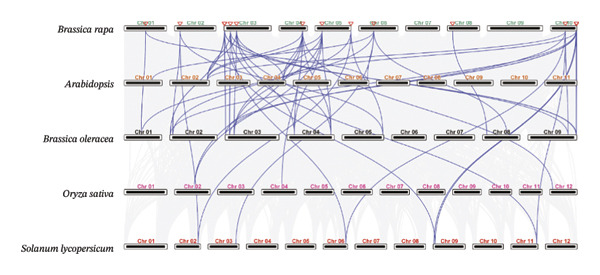
(b)

### 3.5. Duplication Events by a Ratio of Nonsynonymous and Synonymous (Ka/Ks) Value

Typically, Ka/Ks value of genes below 1.0 signifies that these genes are subjected to purifying selection, maintaining functional constraints, while the value of the ratio 1.0 indicates neutral genetic drift. Ratios above one imply positive selection and accelerated evolution [24]. In this study, all six pairs of *BrE2F/DP* paralogs displayed Ka/Ks values < 1.0 (Table 2) suggesting that they have been preserved by strong negative selection. These segmental duplications are guesstimated to have taken place between 10 (Ks = 0.2916) and 19 million years ago (Ks = 0.5708).

### 3.6. Prediction of Cis‐Regulatory Elements

**FIGURE 4:**
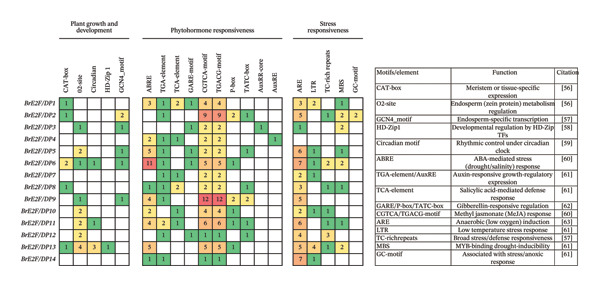
*Cis*‐acting motif and elements identified in the promoter region of BrE2F genes with their function. Numbers indicate the count of elements; the color scale (green–yellow–red) represents low to high counts, respectively.

Analysis of the cis‐regulatory elements in *BrE2F/DP* genes showed the presence of five elements that are engaged in growth and development, 10 responsive to hormonal signals, and five linked to stress response mechanisms. The functions of the motifs and elements are listed in Figure 4. The hormone responsiveness elements were the most abundant. All 14 *BrE2F/DP* promoters contained at least one CGTCA motif and TGACG motif. TGA and ABRE elements were found in 11 and 10 promoters of *BrE2F/DP*, respectively. The promoter analysis also revealed the presence of TCA elements, as well as GARE and P‐box motifs, within *BrE2F/DP* genes. Among the five identified stress‐responsive elements, ARE was observed in all *BrE2F/DP* genes except *BrE2F/DP4*. MBS (a MYB transcription factor binding site) was present in eight genes, while LTR (low‐temperature responsive element) and TC‐rich repeats existed in seven genes. Notably, the GC‐motif was only found in *BrE2F/DP1*. Furthermore, O2‐site was the most abundant among the genes for plant growth and development. Some *BrE2F/DP* genes also had been reported to have circadian regulation, a GCN motif and a CAT box. Except for *BrE2F/DP*13, HD‐Zip 1 was not found in other genes. According to cis‐acting motif and element comparisons, the genes cannot be clearly grouped.

### 3.7. Expression Profiling of E2F/DP Genes in Different *B. rapa* Tissues

**FIGURE 5:**
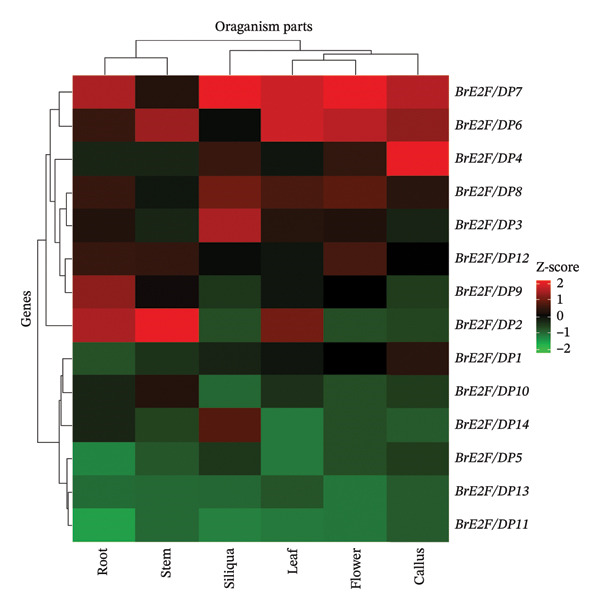
Expression patterns of E2F genes in different *Brassica rapa* tissues. Normalized Log_2_ (FPKM+1) values are plotted against respective tissues. Blocks with red colors’ intensity indicate high intensity of expression of the gene at the transcription level, and green colors represent low accumulation levels.

All *BrE2F/DP* genes were expressed across diverse tissues, including root, stem, siliqua, leaf, flower, and callus (Figure 5), with distinct expression patterns. Among them, *BrE2F/DP3*, *BrE2F/DP4*, *BrE2F/DP6*, *BrE2F/DP7,* and *BrE2F/DP8* exhibited consistently higher expression across all tissues compared to the other family members. However, *BrE2F/DP11*, *BrE2F/DP13,* and *BrE2F/DP5* were hardly expressed. The expression patterns of roots with stems and reproductive organs with leaves were comparable.

### 3.8. Protein‐Protein Interaction, Protein Homology Modeling, and Sequence Similarity

Proteins of genes *BrE2F/DP3*, *4*, *6*, and *7* exhibit strong connections with other proteins and are found as key nodes in the PPI model (Figure 6(a)). The maximum GMQE value was observed in *BrE2F/DP3* (0.74), while the minimum was recorded for *BrE2F/DP12* (0.53). The anticipated structures of BrE2F/DP proteins are included in the SF4. Proteins in each subgroup exhibited a comparable similarity in the quantity of alpha helices and beta sheets. However, the overall sequence similarity among the BrE2F/DP proteins varied across the family. In comparison, proteins belonging to similar categories exhibited a greater similarity percentage (SF5).

FIGURE 6Predicted network of protein–protein interaction (a) and template details (b) of *BrE2F* proteins. Every node of A represents a *BrE2F* protein, and the colored lines denote connections between them. GMQE: global model quality estimation.

**Figure:**
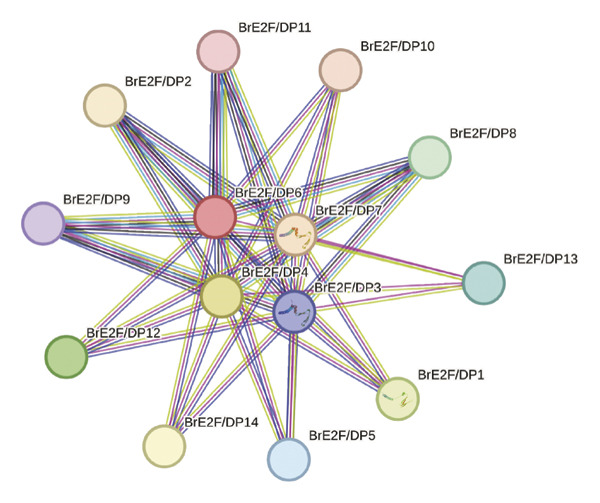
(a)

**Figure:**
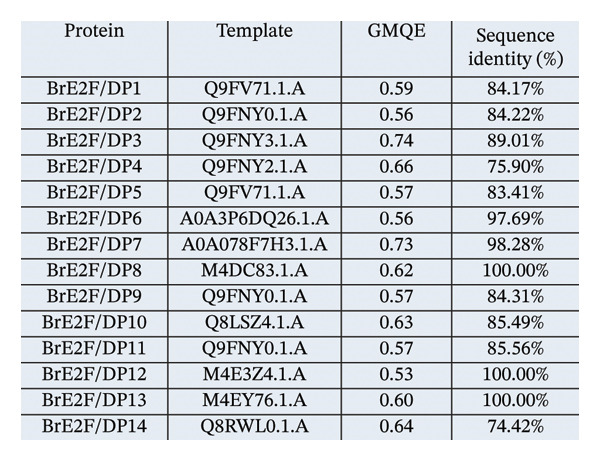
(b)

### 3.9. Prediction of miRNA Target Sites

**FIGURE 7:**
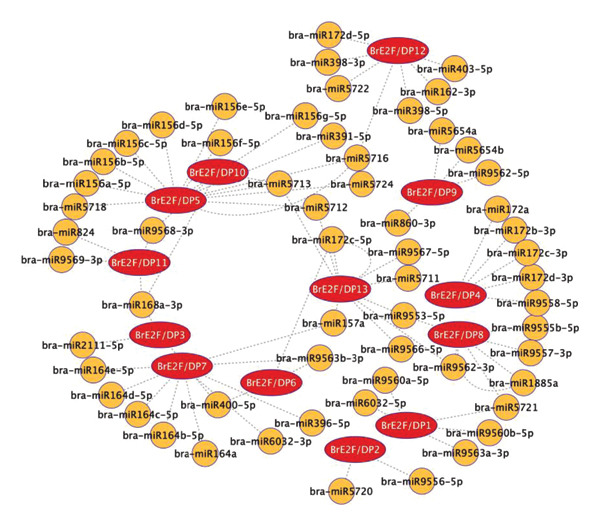
The interaction network between miRNAs and their target BrE2F/DP genes. Red oval circles with gene names, orange round circles are with generic names of microRNAs.

In the latest miRBase release (22.1; http://www.mirbase.org/), 157 mature published miRNAs have been identified in *B. rapa*. The interaction network between miRNAs and their target *BrE2F/DP* genes is shown in Figure 7 including SF6 to show miRNAs, their sequences, targeted *BrE2F/DP* genes, and their mode of translation inhibition. In this study 20–24 bp long total of 76 miRNAs targeting 13 *BrE2F/DP* genes (SF6) were found. Cleavage was the most predominant inhibitory mechanism compared to translation inhibition. Interestingly, *BrE2F/DP14* was not the target of any miRNA (Figure 7). *BrE2F/DP5* was targeted by the highest number of miRNAs (13), followed by *BrE2F/DP13* (10) and *BrE2F/DP7* (9). *BrE2F/DP2* was the least targeted gene by two miRNAs (bra‐miR5720 and bra‐miR9556‐5p). Multiple genes were targeted by the same miRNA. bra‐miR5713 and bra‐miR168a‐3p targeted the highest number of *BrE2F/DP* genes (three genes of each). *BrE2F/DP5*, *BrE2F/DP10*, and *BrE2F/DP13* were the targets of bra‐miR5713, whereas bra‐miR168a‐3p targeted *BrE2F/DP3*, *BrE2F/DP10*, and *BrE2F/DP11*. However, the *BrE2F/DP5* was targeted by several members of the bra‐miR156, bra‐miR5716, and bra‐miR5718 family mRNA, and *BrE2F/DP12* was targeted by bra‐miR398, and bra‐miR6716 (SF6).

## 4. Discussion

### 4.1. *BrE2F/DPs* Evolved by Segmental Duplication Across Chromosomes After *Brassica* Splits From *Arabidopsis*

Gene duplication creates plant evolution by generating diversification of genes [26, 27]. After whole genome duplication, neofunctionalization or subfunctionalization generates divergence in some duplicated genes due to long‐term polyploidization and evolution [28, 29]. Therefore, the number of *E2F/DP* genes (N) varies in different plant species with their genome size (*g*) and ploidy level (*x*). Hence, in *B. rapa* (2x = 16, *g* = 450–580 Mb), it was higher than *Arabidopsis* (*N* = 8, 2x = 10, *g* = 145 Mb) [30], rice (*N* = 9, 2x = 24, *g* = 415–463 Mb) [11], tomato (*N* = 8, 2x = 24, *g* = 907–1000 Mb) [20], barrel medic (*N* = 5, 2x = 16, *g* = 454–526 Mb) [17] but lower than *B. napus* (*N* = 29, 4x = 38, *g* = 1129–1235 Mb) [13], maize (*N* = 21, 2x = 20, *g* = 2292–2716 Mb) [31], wheat (*N* = 27, 6x = 42, *g* = 15,966 Mb) [32], and moso bamboo (*N* = 23, 4x = 48, 2051 Mb) [19] (for genome size, see the literature [33–35]). In consistency with these species, *BrE2F/DP* genes were categorized into E2F, DP, and DEL groups. This division highlights their functional specification within the E2F/DP family and their diverse physiochemical properties in plant biology. Furthermore, our analysis revealed that each phylogenetic group contains representatives from both monocot and dicot species (E2F, DP, and DEL), and the BrE2F/DP proteins exhibited a close phylogenetic relationship with BoE2F/DP and AtE2F/DP proteins. It makes an assumption that these genes arose from a common angiosperm ancestor around 200 million years ago, before the split between monocots and dicots as reported by Wolfe et al. [36], and have since remained evolutionarily conserved in the Crucifer family. From all the pairwise segmental duplication of the BrE2F/DP gene, we can assume that the evolutionary diversification of these genes is mostly by segmental duplication across the chromosome in *B. rapa* and results in diversified functional expressions. However, BrE2F/DP proteins are paired (orthologous) with BoE2F/DP proteins, indicating their functional similarity.

Transcription factor genes were found to be slow‐evolving with low Ka/Ks [37]. Although this family showed diversity in their functions, the Ka/Ks ratios of all duplicated gene pairs were< 1, indicating that these genes evolved slowly under purifying or negative selection and that their functions are relatively conserved in different species. However, the genes *BrE2F/DP2, BrE2F/DP9* and *BrE2F/DP11* evolve comparatively faster. The duplication of genes on a large scale occurred between 10 and 19 million years ago following the divergence of *Brassica* from *Arabidopsis* (12–24 Mys [26, 38]) during the evaluation process, with the most recent duplication estimated to have happened about 10 Mys in *BrE2F1-BrE2F5*. This assumes that the *BrE2F/DP* gene family is an ancient gene family that evolved through segmental duplication during or just after diverging from its ancestors.

### 4.2. Segmental Duplication Arises From Functional Similarity in *Brassica*

The majorities of the identified segmental duplication gene pairs belong to the similar functional group and exhibited a high degree of protein sequence homology and protein structures (e.g., *BrE2F/DP2* with *BrE2F/DP* *9* and *BrE2F/DP* *11*, *BrE2F/DP3* with *BrE2F/DP* *7*, etc.). This suggests that segmentally duplicated genes often maintain similar expression profiles and biological roles due to purifying selection pressures acting to preserve essential functions. This duplication enforces gain and/or loss of exon/intron, exonization/pseudoexonization, and insertion/deletion and tends to evolve into diversiform exon–intron structures with and/or without alteration in amino acid sequence. These lead to the generation of proteins with distinct domain organization and sequence features and result in functionally distinct paralogs [39, 40]. As a result, variations in exon–intron structures were observed among *BrE2F/DP* paralogs, with genes from the same subfamily gradually developing different genetic architectures to suit diverse environments. However, to preserve their original gene structures throughout evolution, the duplicated gene pairs maintained highly conserved exon–intron arrangements. Furthermore, distribution of conserved motifs and domains exhibits similarities within the same subfamily but varies across subfamilies of *BrE2F/DP* genes, suggesting functional diversification across the subfamily.

### 4.3. Structural Divergences of Duplicated Genes Play Important Roles in PPI

The E2F and DP groups contain at least one DBD identified by the presence of a conserved motif sequence “RRIYD,” including the dimerization residue “DNVLE,” to facilitate the formation of homodimers or heterodimers with DP proteins for effective E2F/DP‐DNA binding at the promoter region of a gene [31, 41]. In consistency, a minimum of one DBD was confirmed in each of the *BrE2F/DPs* by the presence of these sequences in motif 1. Additionally, some literature indicated atypical F2F with double DBD to associate with DNA promoter regions independent of DP proteins [1, 42]. However, in *BrE2F/DPs*, all the DEL group members are identified as atypical due to having two DBDs. The E2F subfamily binds to the DP subfamily with a DD domain to produce the E2F‐DP complex, which binds to the C‐terminal domain of retinoblastoma (Rb) by the CC‐MB domains of the E2F‐DP complex to regulate the G1–S transition pathway [43]. The interaction between E2F and retinoblastoma (Rb) to produce the RBC‐E2F‐DP complex is confirmed by the presence of CC‐MB domains adjacent to the TD (transactivation domain) of all E2Fs and DD domains in some members (*BrE2F/DP3 and 7*) of the DP subfamily. These result in the protein structural similarities and strong PPI in the members of the DP group, although all the proteins interact with each other due to the presence of DBDs.

### 4.4. Multifaceted Role of BrE2F/DP Genes in *B. rapa* Growth and Development

Although in all eukaryotes, E2F/DP family proteins regulate cell‐cycle operating gene expression as transcription factors, their localization varied in plant species. *PvE2F/DP* genes are located both in the nucleus and in other organelles [44], and they function not only in cell cycle regulation but also in stress response. Proteins with similar subcellular localization often predict their functional association by mutual PPIs [45]. The localization of plant E2Fs in the nucleus has been reported to be mediated by their interaction with DPs or other regulatory proteins [46]. Subcellular localization of all BrE2F/DP proteins in the nucleus indicates their functional association with interaction in cell cycle‐regulating gene expression. However, *BrE2F/DP* genes varied in their roles in growth and developmental processes in plants. Strong expression of *BrE2F/DP3, BrE2F/DP4, BrE2F/DP6, BrE2F/DP7,* and *BrE2F/DP8* across almost all types of tissues, indicating their potential roles in fundamental cellular functions. However, *BrE2F/DP5, BrE2F/DP11,* and *BrE2F/DP13* had very low or no expression across all tissues. This is either because of their functional redundancy or expression specificity at definite plant growth stages or under specific environmental stimuli. Nevertheless, generic expression of the DP group was always higher compared to the E2F and DEL groups. As a heterodimeric partner, E2F/DP complex formation is necessary for enhancing the DNA‐binding activity and transcriptional specificity of E2F members [8, 47]. In cell cycle regulation, DPs act as a key regulatory node for influencing the formation and stability of functional E2F/DP complexes, thereby promoting the transcription of genes required for DNA replication. Such dominance in expression could have a broad influence on downstream regulatory genes and thus on various physiological and developmental processes [47]. Tissue‐specific expression patterns suggest that BrE2F/DP transcription factors not only control the cell cycle but also support organ‐specific differentiation programs. For example, *BrE2F/DP14* showed higher expression in the siliqua, potentially indicating its role in reproductive tissue development, while *BrE2F/DP2* was primarily expressed in roots, stems, and leaves, suggesting a function in vegetative tissue development. Collectively, the varied expression suggests the multifaceted role of *BrE2F/DP* genes in *B. rapa* plant organogenesis, supporting earlier findings in *A. thaliana* and other plant species. Further functional characterization of these genes can elucidate the precise roles of these transcription factors in regulating plant growth.

### 4.5. BrE2F/DP Genes Are the Targets of Stress Responsive miRNAs

miRNAs are key regulators of numerous plant biological functions, including development, growth, nutrient uptake, signaling, flowering, and reactions to both biotic and abiotic stress factors. It was reported that clubroot resistance genes (Bra019412 and Bra019410) in *B. rapa* were downregulated by bra‐miR1885a and bra‐miR1885b during early *Plasmodiophora brassicae* infection. bra‐miR1885a and bra‐miR1885b were anticipated to suppress the expression of target genes by interfering with translation and promoting cleavage mechanisms, respectively [48]. The same miRNA can influence multiple target genes within a gene family and regulate the target genes under biotic and abiotic stress [49, 50]. The family of bra‐miR156, bra‐miR398, bra‐miR1885, bra‐miR5716, and bra‐miR5718 was reported to be sensitive to heat stress in *B. rapa* [49]. In this study most of them targeted *BrE2F/DP5* and *BrE2F/DP12*. Additionally, the BrE2F/DP1 and BrE2F/DP8 were also targeted by the miRNA responses under heat stress. Therefore, we can suspect that these genes may be involved in abiotic stress resistance.

### 4.6. Multifunctional Roles Attributed to *BrE2F/DP*

It was well reported that plant transcription factor genes respond to multiple environmental stimuli [51]. Therefore, a diverse array of cis‐regulatory elements associated with growth, hormone, and metabolic regulation under various stress conditions was detected in the promoter regions of BrE2F/DP genes. In total, 10 motifs were found to be related to phytohormone signaling pathways. Among them, CGTCA and TGACG motifs were in all the *BrE2F/DP*, which are responsive to methyl jasmonate (MeJA) and jasmonic acid (JA), respectively. These elements are typically the most potential defense hormones to boost up the plant resistance against biotic and abiotic stressors in crosstalk with other plant hormones (IAA, GA, ABA, ET, SA, and BR) and metabolites to regulate numerous enzymes and proteins [52]. Therefore, the detection of elements for abscisic acid (ABREs) and salicylic acid (TCA) and motifs for gibberellin (GARE, TATC‐box, and P‐box) and auxin‐responsive (AuxRR) in *BrE2F/DP* suggesting that these may be stimulated by environmental stress to activate defense mediated by JA‐ and MeJA‐mediated signaling pathways. Additionally, among the stress‐responsive elements, the presence of ARE (adenylate–uridylate‐rich element/antioxidant response elements) in nearly all *BrE2F/DP* promoters indicates a broad responsiveness to antioxidative defense mechanisms under anaerobic or hypoxic conditions. Under oxygen stress, SNF1‐related protein kinase‐1 (SnRK1) is activated and directly phosphorylates E2F transcription factors to coordinate cell proliferation and vegetative growth, enabling plants to adapt to dynamic environmental conditions [53]. Furthermore, the detection of MBS (MYB binding sites involved in drought inducibility) and LTR (long terminal repeat) retrotransposons suggests potential regulation of these genes in response to water deficit and cold stress [54, 55]. However, the identification of motifs and elements related to plant growth and development aligns with the established function of E2F/DP transcription factors in regulating cell division and organogenesis. Therefore, this diversity in cis‐element composition of *RrE2F/DP* transcription factors aligns not only with cell cycle progression but also with integrated developmental and environmental signals for adaptation under biotic and abiotic stresses. Nevertheless, further experimental validation will help to elucidate the multifunctional roles of these elements in regulating *E2F/DP* gene expression in *B. rapa*.

In conclusion, the results of this study provided extensive information about the structural and functional differences in E2F‐DP family members based on their sequence of genes and proteins, their structure, protein interaction, their expression, and miRNA targets in *B. rapa* as a member of the *Brassica* subspecies. Their involvement in defense mechanisms against different stressors was the most important observation, which needs further research on the precise roles of each member and their interactions for resistance mechanisms against environmental stress. This can contribute to the potential advancement of *Brassica* stress breeding programs.

## Funding

The authors received no specific funding for this work.

## Conflicts of Interest

The authors declare no conflicts of interest.

## Supporting Information

Additional supporting information can be found online in the Supporting Information section.

## Supporting information


**Supporting Information 1** SF1. Sequence Alignment of E2F/DP Proteins.

**Supporting Information 2** SF2. Sequence identity (%) among the 14 E2F/DP proteins of *B. rapa*.

**Supporting Information 3** SF3: Syntenic pairs of E2F proteins.

**Supporting Information 4** SF4: Structure of BrE2F/DP proteins.

**Supporting Information 5** SF5. Sequence identity of BrE2F/DP proteins. GMQE: global model quality estimation.

**Supporting Information 6** SF6: Details of the miRNAs and their target *BrE2F/DP* genes.

## Data Availability

The data that support the findings of this study are available on request from the corresponding author. The data are not publicly available due to privacy or ethical restrictions.
