# Supplementary material for: Genome Wide Identification and Characterization of BrE2F Family Gene of Brassica rapa
Source: Int J Genomics. 2026 Jun 15;2026:7106391. doi: 10.1155/ijog/7106391 (PMC13269648; doi:10.1155/ijog/7106391)
Supplement: Supplementary file 1 — Supporting Information 1 SF1. Sequence Alignment of E2F/DP Proteins. [file IJOG-2026-7106391-s006.doc]

| **SF1. Sequence Alignment of E2F/DP Proteins.** |
| --- |
| 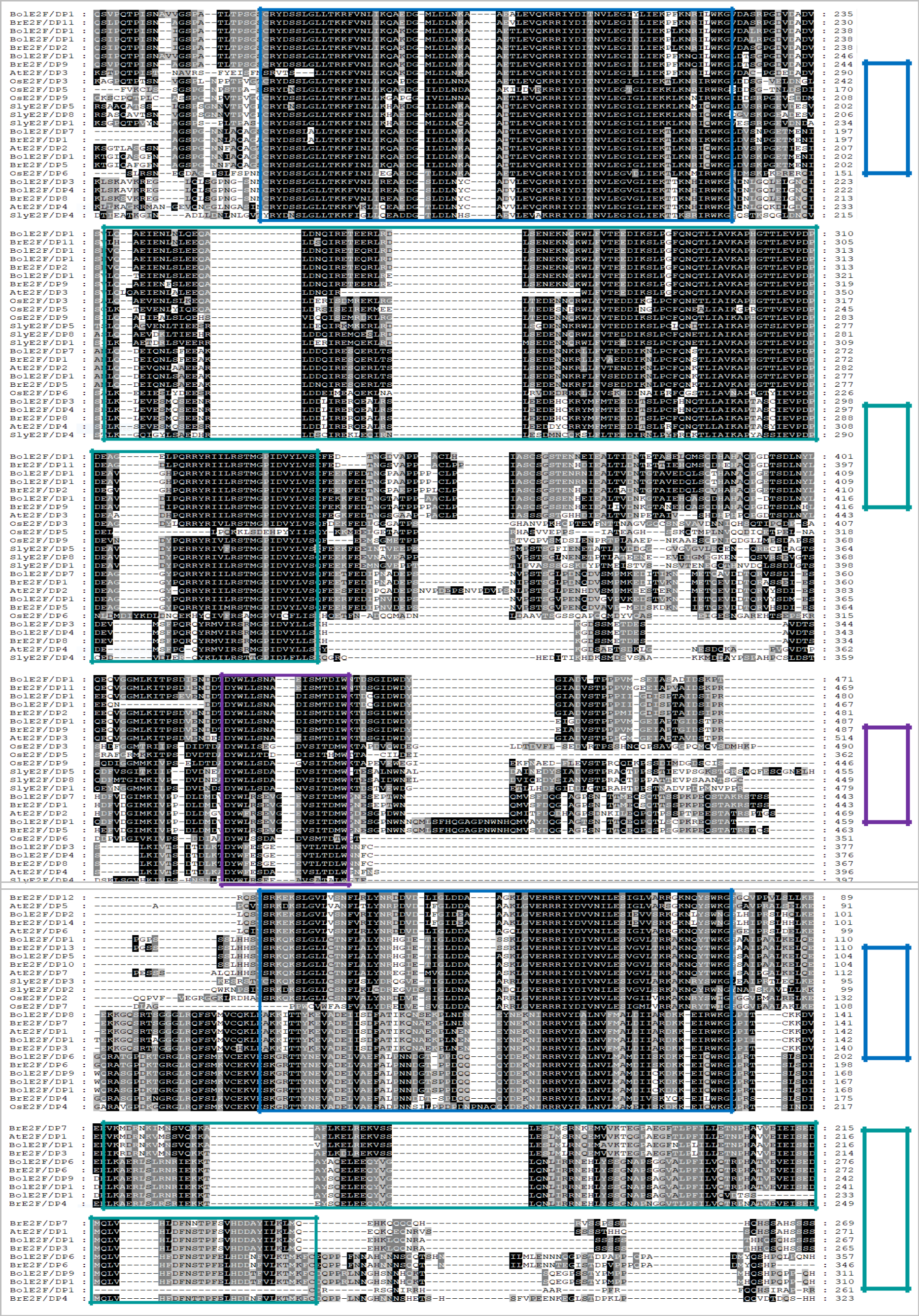 DNA binding  Dimerization  RB binding  DNA binding  Dimerization |
