# Supplementary material for: Genome Wide Identification and Characterization of BrE2F Family Gene of Brassica rapa
Source: Int J Genomics. 2026 Jun 15;2026:7106391. doi: 10.1155/ijog/7106391 (PMC13269648; doi:10.1155/ijog/7106391)
Supplement: Supplementary file 2 — Supporting Information 2 SF2. Sequence identity (%) among the 14 E2F/DP proteins of B. rapa. [file IJOG-2026-7106391-s005.docx]

| **SF2. Sequence identity (%) among the 14 E2F/DP proteins of *B. rapa*** | | | | | | | | | | | | | | |
| --- | --- | --- | --- | --- | --- | --- | --- | --- | --- | --- | --- | --- | --- | --- |
| **Protein** | ***BrE2F/DP1*** | ***BrE2F/DP2*** | ***BrE2F/DP3*** | ***BrE2F/DP4*** | ***BrE2F/DP5*** | ***BrE2F/DP6*** | ***BrE2F/DP7*** | ***BrE2F/DP8*** | ***BrE2F/DP9*** | ***BrE2F/DP10*** | ***BrE2F/DP11*** | ***BrE2F/DP12*** | ***BrE2F/DP13*** | ***BrE2F/DP14*** |
| ***BrE2F/DP1*** | 100 |  |  |  |  |  |  |  |  |  |  |  |  |  |
| ***BrE2F/DP2*** | 50 | 100 |  |  |  |  |  |  |  |  |  |  |  |  |
| ***BrE2F/DP3*** | 28.77 | 28.74 | 100 |  |  |  |  |  |  |  |  |  |  |  |
| ***BrE2F/DP4*** | 30.14 | 34.62 | 50.52 | 100 |  |  |  |  |  |  |  |  |  |  |
| ***BrE2F/DP5*** | 80.6 | 49.65 | 28.77 | 30.14 | 100 |  |  |  |  |  |  |  |  |  |
| ***BrE2F/DP6*** | 37.5 | 34.62 | 53.92 | 71.5 | 36.51 | 100 |  |  |  |  |  |  |  |  |
| ***BrE2F/DP7*** | 30.14 | 29.89 | 86.97 | 51.04 | 30.14 | 53.43 | 100 |  |  |  |  |  |  |  |
| ***BrE2F/DP8*** | 47.3 | 60.53 | 28 | 28.95 | 46.27 | 30.49 | 28 | 100 |  |  |  |  |  |  |
| ***BrE2F/DP9*** | 49.42 | 83.33 | 29.89 | 37.66 | 49.06 | 37.66 | 31.03 | 57.14 | 100 |  |  |  |  |  |
| ***BrE2F/DP10*** | 43.28 | 43.48 | 36.17 | 33.33 | 44.78 | 40 | 36.17 | 45.45 | 44.78 | 100 |  |  |  |  |
| ***BrE2F/DP11*** | 51.05 | 82.86 | 38.46 | 33.33 | 51.3 | 33.85 | 29.89 | 47.24 | 82.06 | 40.45 | 100 |  |  |  |
| ***BrE2F/DP12*** | 27.92 | 29.23 | 36.67 | 32.47 | 31.53 | 36.67 | 36.67 | 36.99 | 35.45 | 50.38 | 29.74 | 100 |  |  |
| ***BrE2F/DP13*** | 40.3 | 40.58 | 36.17 | 30.95 | 41.79 | 36.67 | 36.17 | 42.42 | 41.79 | 84.53 | 40.58 | 43.11 | 100 |  |
| ***BrE2F/DP14*** | 32.43 | 35.82 | 33.33 | 38.78 | 34.33 | 34.69 | 33.33 | 33.33 | 35.82 | 43.48 | 35.82 | 58.08 | 44.97 | 100 |
