# Supplementary material for: Genome Wide Identification and Characterization of BrE2F Family Gene of Brassica rapa
Source: Int J Genomics. 2026 Jun 15;2026:7106391. doi: 10.1155/ijog/7106391 (PMC13269648; doi:10.1155/ijog/7106391)
Supplement: Supplementary file 3 — Supporting Information 3 SF3: Syntenic pairs of E2F proteins. [file IJOG-2026-7106391-s004.docx]

| **SF3: Syntenic pairs of E2F proteins** | | | | | |
| --- | --- | --- | --- | --- | --- |
| **Gene Name** | **Gene ID** | **Syntenyc Pair** | **Species** | **No of Syntenic Pairs** | |
| *BrE2F/DP1* | Bra002398 | Cla97C02G048470.2 | Arabidopsis |  | |
|  | Bra002398 | Solyc06T002260.1.ITAG5.0 | Tomato | Cabbage | 36 |
|  | Bra002398 | Solyc11T002240.1.ITAG5.0 | Tomato | Arabidopsis | 12 |
|  | Bra002398 | BolC03g011370.2J.m1 | Cabbage | Rice | 4 |
|  | Bra002398 | BolC09g050820.2J.m1 | Cabbage | Tomato | 10 |
| *BrE2F/DP2* | Bra005305 | Cla97C02G048470.2 | Arabidopsis | **Total** | **62** |
|  | Bra005305 | Cla97C04G077210.2 | Arabidopsis |  |  |
|  | Bra005305 | LOC_Os02g33430.1.MSUv7.0 | Rice |  |  |
|  | Bra005305 | BolC03g021280.2J.m1 | Cabbage |  |  |
|  | Bra005305 | BolC04g012950.2J.m1 | Cabbage |  |  |
|  | Bra005305 | BolC04g061350.2J.m1 | Cabbage |  |  |
| *BrE2F/DP3* | Bra005726 | Solyc09T000476.1.ITAG5.0 | Tomato |  |  |
|  | Bra005726 | BolC02g001130.2J.m1 | Cabbage |  |  |
|  | Bra005726 | BolC03g000910.2J.m1 | Cabbage |  |  |
|  | Bra005726 | BolC03g001350.2J.m1 | Cabbage |  |  |
|  | Bra005726 | BolC09g068430.2J.m1 | Cabbage |  |  |
|  | Bra005726 | BolC09g069040.2J.m1 | Cabbage |  |  |
| *BrE2F/DP4* | Bra005763 | BolC02g001130.2J.m1 | Cabbage |  |  |
|  | Bra005763 | BolC03g000910.2J.m1 | Cabbage |  |  |
|  | Bra005763 | BolC03g001350.2J.m1 | Cabbage |  |  |
|  | Bra005763 | BolC09g068430.2J.m1 | Cabbage |  |  |
|  | Bra005763 | BolC09g069040.2J.m1 | Cabbage |  |  |
| *BrE2F/DP5* | Bra006615 | Cla97C02G048470.2 | Arabidopsis |  |  |
|  | Bra006615 | Solyc06T002260.1.ITAG5.0 | Tomato |  |  |
|  | Bra006615 | Solyc11T002240.1.ITAG5.0 | Tomato |  |  |
|  | Bra006615 | BolC03g011370.2J.m1 | Cabbage |  |  |
|  | Bra006615 | BolC09g050820.2J.m1 | Cabbage |  |  |
| *BrE2F/DP6* | Bra009530 | Cla97C04G076100.2 | Arabidopsis |  |  |
|  | Bra009530 | Solyc09T000226.1.ITAG5.0 | Tomato |  |  |
|  | Bra009530 | BolC02g001130.2J.m1 | Cabbage |  |  |
|  | Bra009530 | BolC03g001350.2J.m1 | Cabbage |  |  |
|  | Bra009530 | BolC09g068430.2J.m1 | Cabbage |  |  |
| *BrE2F/DP7* | Bra009586 | Solyc09T000476.1.ITAG5.0 | Tomato |  |  |
|  | Bra009586 | BolC03g000910.2J.m1 | Cabbage |  |  |
|  | Bra009586 | BolC09g069040.2J.m1 | Cabbage |  |  |
| *BrE2F/DP8* | Bra014096 | BolC08g006010.2J.m1 | Cabbage |  |  |
|  | Bra017268 | Cla97C02G048470.2 | Arabidopsis |  |  |
|  | Bra017268 | Cla97C04G077210.2 | Arabidopsis |  |  |
|  | Bra017268 | LOC_Os02g33430.1.MSUv7.0 | Rice |  |  |
|  | Bra017268 | LOC_Os04g33950.1.MSUv7.0 | Rice |  |  |
|  | Bra017268 | LOC_Os12g06200.1.MSUv7.0 | Rice |  |  |
|  | Bra017268 | BolC03g021280.2J.m1 | Cabbage |  |  |
|  | Bra017268 | BolC04g012950.2J.m1 | Cabbage |  |  |
|  | Bra017268 | BolC04g061350.2J.m1 | Cabbage |  |  |
| *BrE2F/DP10* | Bra018080 | Cla97C06G126260.1 | Arabidopsis |  |  |
|  | Bra018080 | Cla97C01G013130.2 | Arabidopsis |  |  |
|  | Bra018080 | Solyc03T002633.1.ITAG5.0 | Tomato |  |  |
|  | Bra018080 | BolC01g030510.2J.m1 | Cabbage |  |  |
|  | Bra018080 | BolC08g032240.2J.m1 | Cabbage |  |  |
| *BrE2F/DP11* | Bra023047 | Cla97C02G048470.2 | Arabidopsis |  |  |
|  | Bra023047 | Cla97C04G077210.2 | Arabidopsis |  |  |
|  | Bra023047 | BolC03g021280.2J.m1 | Cabbage |  |  |
|  | Bra023047 | BolC04g012950.2J.m1 | Cabbage |  |  |
|  | Bra023047 | BolC04g061350.2J.m1 | Cabbage |  |  |
| *BrE2F/DP12* | Bra023497 | Solyc02T002300.1.ITAG5.0 | Tomato |  |  |
|  | Bra023497 | BolC02g006670.2J.m1 | Cabbage |  |  |
|  | Bra023497 | BolC05g062280.2J.m1 | Cabbage |  |  |
| *BrE2F/DP13* | Bra033767 | Cla97C06G126260.1 | Arabidopsis |  |  |
|  | Bra033767 | BolC01g030510.2J.m1 | Cabbage |  |  |
| *BrE2F/DP14* | Bra039127 | Solyc02T002300.1.ITAG5.0 | Tomato |  |  |
|  | Bra039127 | BolC02g006670.2J.m1 | Cabbage |  |  |
|  | Bra039127 | BolC05g062280.2J.m1 | Cabbage |  |  |
|  |  |  |  |  |  |
