# Supplementary figures and images for: Genome Wide Identification and Characterization of BrE2F Family Gene of Brassica rapa
Source: Int J Genomics. 2026 Jun 15;2026:7106391. doi: 10.1155/ijog/7106391 (PMC13269648; doi:10.1155/ijog/7106391)

| **SF4: Structure of BrE2F/DP proteins.** |
| --- |
| **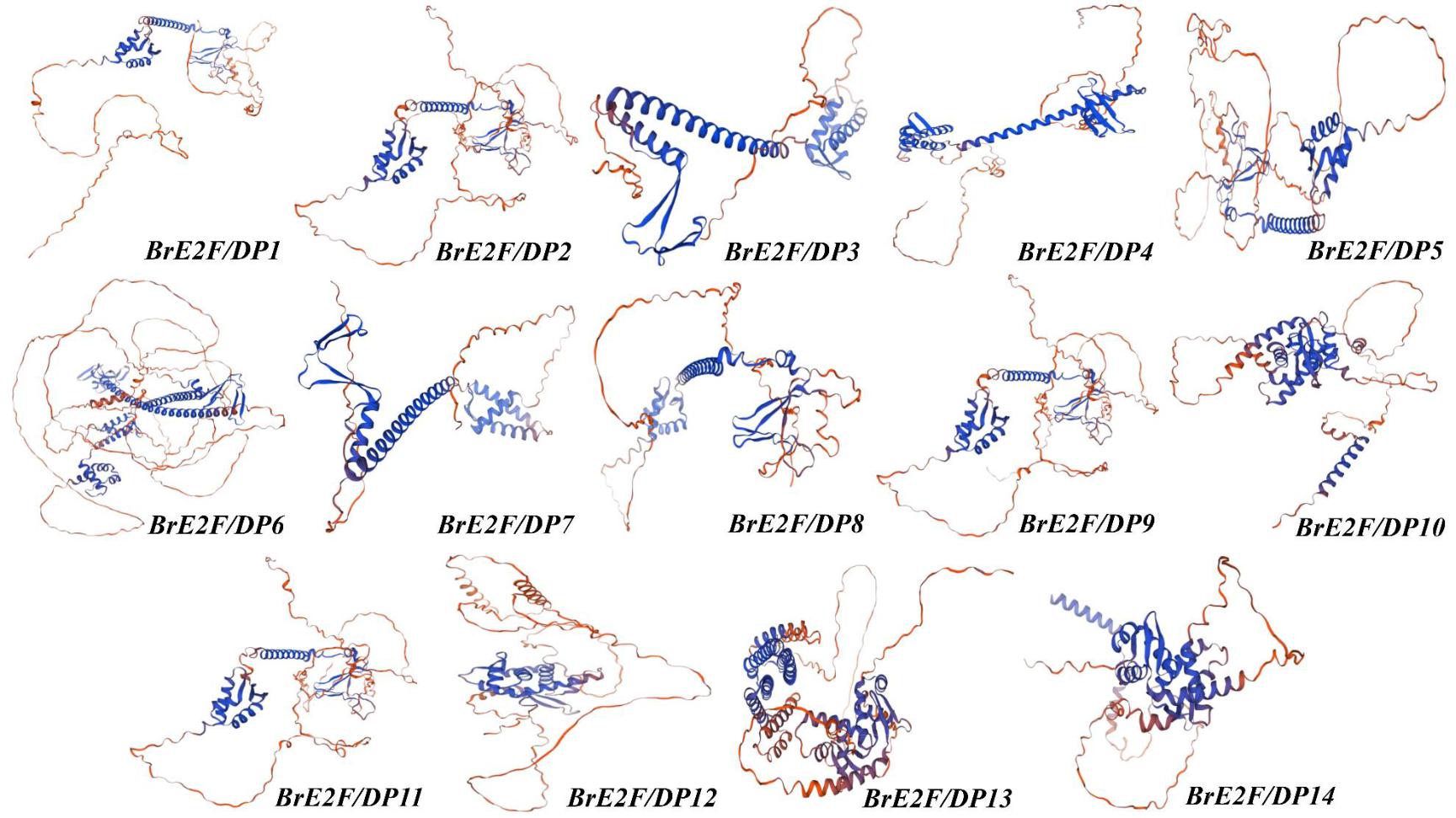** |

Supplement: Supplementary file 4 — Supporting Information 4 SF4: Structure of BrE2F/DP proteins. [file IJOG-2026-7106391-s003.doc]
