# Supplementary material for: Genome Wide Identification and Characterization of BrE2F Family Gene of Brassica rapa
Source: Int J Genomics. 2026 Jun 15;2026:7106391. doi: 10.1155/ijog/7106391 (PMC13269648; doi:10.1155/ijog/7106391)
Supplement: Supplementary file 5 — Supporting Information 5 SF5. Sequence identity of BrE2F/DP proteins. GMQE: global model quality estimation. [file IJOG-2026-7106391-s002.doc]

**SF5. Sequence identity of BrE2F/DP proteins.**

| **Protein** | **Template** | **GMQE** | **Seq Identity** |
| --- | --- | --- | --- |
|  |  |  |  |
| *BrE2F/DP1* | [Q9FV71.1.A](https://swissmodel.expasy.org/repository/uniprot/Q9FV71?model=AF-Q9FV71-F1-model-v4) | 0.59 | 84.17% |
| *BrE2F/DP2* | [Q9FNY0.1.A](https://swissmodel.expasy.org/repository/uniprot/Q9FNY0?model=AF-Q9FNY0-F1-model-v4) | 0.56 | 84.22% |
| *BrE2F/DP3* | [Q9FNY3.1.A](https://swissmodel.expasy.org/repository/uniprot/Q9FNY3?model=AF-Q9FNY3-F1-model-v4) | 0.74 | 89.01% |
| *BrE2F/DP4* | [Q9FNY2.1.A](https://swissmodel.expasy.org/repository/uniprot/Q9FNY2?model=AF-Q9FNY2-F1-model-v4) | 0.66 | 75.90% |
| *BrE2F/DP5* | [Q9FV71.1.A](https://swissmodel.expasy.org/repository/uniprot/Q9FV71?model=AF-Q9FV71-F1-model-v4) | 0.57 | 83.41% |
| *BrE2F/DP6* | [A0A3P6DQ26.1.A](https://swissmodel.expasy.org/repository/uniprot/A0A3P6DQ26?model=AF-A0A3P6DQ26-F1-model-v4) | 0.56 | 97.69% |
| *BrE2F/DP7* | [A0A078F7H3.1.A](https://swissmodel.expasy.org/repository/uniprot/A0A078F7H3?model=AF-A0A078F7H3-F1-model-v4) | 0.73 | 98.28% |
| *BrE2F/DP8* | [M4DC83.1.A](https://swissmodel.expasy.org/repository/uniprot/M4DC83?model=AF-M4DC83-F1-model-v4) | 0.62 | 100.00% |
| *BrE2F/DP9* | [Q9FNY0.1.A](https://swissmodel.expasy.org/repository/uniprot/Q9FNY0?model=AF-Q9FNY0-F1-model-v4) | 0.57 | 84.31% |
| *BrE2F/DP10* | [Q8LSZ4.1.A](https://swissmodel.expasy.org/repository/uniprot/Q8LSZ4?model=AF-Q8LSZ4-F1-model-v4) | 0.63 | 85.49% |
| *BrE2F/DP11* | [Q9FNY0.1.A](https://swissmodel.expasy.org/repository/uniprot/Q9FNY0?model=AF-Q9FNY0-F1-model-v4) | 0.57 | 85.56% |
| *BrE2F/DP12* | [M4E3Z4.1.A](https://swissmodel.expasy.org/repository/uniprot/M4E3Z4?model=AF-M4E3Z4-F1-model-v4) | 0.53 | 100.00% |
| *BrE2F/DP13* | [M4EY76.1.A](https://swissmodel.expasy.org/repository/uniprot/M4EY76?model=AF-M4EY76-F1-model-v4) | 0.60 | 100.00% |
| *BrE2F/DP14* | [Q8RWL0.1.A](https://swissmodel.expasy.org/repository/uniprot/Q8RWL0?model=AF-Q8RWL0-F1-model-v4) | 0.64 | 74.42% |
|  |  |  |  |
