# Supplementary material for: Genome Wide Identification and Characterization of BrE2F Family Gene of Brassica rapa
Source: Int J Genomics. 2026 Jun 15;2026:7106391. doi: 10.1155/ijog/7106391 (PMC13269648; doi:10.1155/ijog/7106391)
Supplement: Supplementary file 6 — Supporting Information 6 SF6: Details of the miRNAs and their target BrE2F/DP genes. [file IJOG-2026-7106391-s001.doc]

| **Supplementary Table ST6: Details of the miRNAs and their target BrE2F/DP genes** | | | | | | | | | | | |
| --- | --- | --- | --- | --- | --- | --- | --- | --- | --- | --- | --- |
| **miRNA_Acc.** | **Target_Acc.** | **Expectation** | **miRNA_start** | **miRNA_end** | **Target_start** | **Target_end** | **miRNA_aligned_fragment** | **alignment** | **Target_aligned_fragment** | **Inhibition** | **Multiplicity** |
| bra-miR1885a | BrE2F/DP8 | 3.5 | 1 | 22 | 1946 | 1967 | CAUCAAUGAAAGGUAUGAUUCC | ..:: ::.::::.:::::: | CUGGUCUUAUCUUUUAUUGAUC | Cleavage | 2 |
| bra-miR396-5p | BrE2F/DP7 | 3.5 | 1 | 21 | 849 | 869 | UUCCACAGCUUUCUUGAACUU | : :::::::: ::::::: | ACCUGCAAGAAAGAUGUGGAA | Cleavage | 1 |
| bra-miR400-5p | BrE2F/DP3 | 3.5 | 1 | 21 | 837 | 857 | UAUGAGAGUAUUAUAAGUCAC | ::. :::.:::::::.:: | UGGAUAUAUGAUACUCUUAUU | Cleavage | 1 |
| bra-miR860-3p | BrE2F/DP9 | 3.5 | 1 | 21 | 3047 | 3067 | UCAAUACAUUGGACUACAUAU | ::::.:::.::::: ::: | UGAUGUGGUCUAAUGUUUUGC | Cleavage | 1 |
| bra-miR9553-5p | BrE2F/DP8 | 3.5 | 1 | 22 | 552 | 573 | UACAAAGCUGAAGCUAAUUAUG | : ::: :::.:::::: ::::. | CUUAAGUAGUUUCAGCAUUGUG | Cleavage | 1 |
| bra-miR9567-5p | BrE2F/DP13 | 3.5 | 1 | 21 | 3589 | 3609 | UAAACAACACAUAUAGUUUGC | :..:::::::::::: :: | AAAGGCUAUAUGUGUUGCUUC | Cleavage | 1 |
| bra-miR157a | BrE2F/DP13 | 4 | 1 | 21 | 1038 | 1058 | UUGACAGAAGAUAGAGAGCAC | :::::.:::.: :: :::: | AAGCUCUUUAUUUGCUCUCAA | Cleavage | 1 |
| bra-miR172c-5p | BrE2F/DP13 | 4 | 1 | 21 | 3831 | 3851 | GCAUCAUCAUCAAGAUUCAGA | : .::. :::::::::::. | AGUUGAUUGUGAUGAUGAUGU | Cleavage | 2 |
| bra-miR5718 | BrE2F/DP5 | 4 | 1 | 22 | 2295 | 2316 | UCAGAACCAAACACAGAACAAG | .: : :.: :::::: :::::: | UUAGGUUUCUGUUUGUUUCUGA | Cleavage | 1 |
| bra-miR6032-5p | BrE2F/DP1 | 4 | 1 | 21 | 369 | 388 | AACAUGGAGCAUCAACAGAUC | :.:.::: :.::.:.:::::: | GGUUUGU-GGUGUUUCAUGUU | Cleavage | 1 |
| bra-miR9555b-5p | BrE2F/DP8 | 4 | 1 | 21 | 698 | 718 | UGUAAUUGCGGGGUUCUAAGC | .::. .:..:.:::::::.: | CUUUGAGAUUCUGCAAUUAUA | Cleavage | 1 |
| bra-miR9563a-3p | BrE2F/DP1 | 4 | 1 | 21 | 3188 | 3208 | UAAAAGUUAAGAGACAAGUUA | ::. : :::.:::.:::::. | AAAUCUCUCUUUUAGCUUUUG | Cleavage | 1 |
| bra-miR9563b-3p | BrE2F/DP7 | 4 | 1 | 22 | 262 | 283 | AAAUUAAGAGAUGAAUUCUUAC | .:: ::.:::::: :.::: | UAUGGAUUUUAUCUCUCAGUUU | Cleavage | 1 |
| bra-miR168a-3p | BrE2F/DP11 | 4.5 | 1 | 21 | 383 | 403 | CCCGCCUUGUAUCAAGUGAAU | :::.::: :::..::: :: | UCUCAUUUGUUACGGGGCCGG | Cleavage | 1 |
| bra-miR172a | BrE2F/DP4 | 4.5 | 1 | 21 | 28 | 48 | AGAAUCUUGAUGAUGCUGCAU | : :::::: :::::::: | CACAACCAUCAUGAAGAUUCU | Cleavage | 1 |
| bra-miR172b-3p | BrE2F/DP4 | 4.5 | 1 | 21 | 28 | 48 | AGAAUCUUGAUGAUGCUGCAU | : :::::: :::::::: | CACAACCAUCAUGAAGAUUCU | Cleavage | 1 |
| bra-miR172c-3p | BrE2F/DP4 | 4.5 | 1 | 21 | 28 | 48 | AGAAUCUUGAUGAUGCUGCAG | : :::::: :::::::: | CACAACCAUCAUGAAGAUUCU | Cleavage | 1 |
| bra-miR2111-5p | BrE2F/DP3 | 4.5 | 1 | 21 | 455 | 475 | UAAUCUGCAUCCUGGGGUUUA | .:..::::: ::::::: : | GUGAUUCCAGGUUGCAGAUGA | Translation | 1 |
| bra-miR403-5p | BrE2F/DP12 | 4.5 | 1 | 22 | 1150 | 1171 | UGUUUUGUGCGUGAAUCUAAUU | :.:.::: .:::::.::: | CUAUGGGUUCUUGCACAGAACU | Cleavage | 1 |
| bra-miR5711 | BrE2F/DP13 | 4.5 | 1 | 21 | 1188 | 1208 | UGUUUUGUGGGUUUCUACCGA | .:: ::: .::::::: ::: | CUGGGAGAUGCCCACAAUACA | Cleavage | 1 |
| bra-miR5712 | BrE2F/DP5 | 4.5 | 1 | 21 | 3094 | 3114 | AAUAUUAAUAUAAUUGGUGAG | .:::..:::::: : :::.:: | UUCAUUAAUUAUCUGAAUGUU | Cleavage | 1 |
| bra-miR5712 | BrE2F/DP13 | 4.5 | 1 | 21 | 3720 | 3740 | AAUAUUAAUAUAAUUGGUGAG | :: . :::.:::: ::::: | UGCAAUUAUUGUAUUCAUAUU | Cleavage | 1 |
| bra-miR5721 | BrE2F/DP1 | 4.5 | 1 | 21 | 1892 | 1912 | AAAAAUGGAGUGAGAAAUGGA | :..::::::...:.:. :::: | UUUAUUUCUUGUUUCGGUUUU | Cleavage | 1 |
| bra-miR6032-3p | BrE2F/DP7 | 4.5 | 1 | 22 | 1281 | 1302 | UCUGCUGGUCGUUCCAUGUUAA | : :: : ::: :::::::::: | UGAAGACUGAAGGACCAGCAGA | Translation | 1 |
| bra-miR9557-3p | BrE2F/DP8 | 4.5 | 1 | 21 | 742 | 762 | GCUGAGUUGGAACGCAAAAUC | :::: .:::....::::: | UUUUUUUUGUUUUGGCUCAGG | Cleavage | 1 |
| bra-miR9560a-5p | BrE2F/DP1 | 4.5 | 1 | 24 | 1538 | 1561 | ACAGGUGGUGGAACAAAUAUGAGU | :. :.:: :::::: ..:::::: | AUGCGUAGAUGUUCCUUUACCUGU | Cleavage | 1 |
| bra-miR9560b-5p | BrE2F/DP1 | 4.5 | 1 | 24 | 1538 | 1561 | ACAGGUGGUGGAACAAAUAUGAGU | :. :.:: :::::: ..:::::: | AUGCGUAGAUGUUCCUUUACCUGU | Cleavage | 1 |
| bra-miR9568-3p | BrE2F/DP11 | 4.5 | 1 | 21 | 2055 | 2075 | UCAUCGUAAGAGAUCUGCAUU | .::::: : :::::.::::: | GAUGCAAAAGUCUUAUGAUGA | Cleavage | 1 |
| bra-miR9569-3p | BrE2F/DP11 | 4.5 | 1 | 24 | 2713 | 2736 | ACACAGGAACAAUACUAACUCAUU | :: :::::::: ::.::: : | UAACAGGUAGUAUUGGUCUUGUAU | Cleavage | 1 |
| bra-miR156a-5p | BrE2F/DP5 | 5 | 1 | 20 | 399 | 418 | UGACAGAAGAGAGUGAGCAC | :::::::.:. :::.:: .: | GUGCUCAUUUACUUUUGCUA | Translation | 1 |
| bra-miR156b-5p | BrE2F/DP5 | 5 | 1 | 20 | 399 | 418 | UGACAGAAGAGAGUGAGCAC | :::::::.:. :::.:: .: | GUGCUCAUUUACUUUUGCUA | Translation | 1 |
| bra-miR156c-5p | BrE2F/DP5 | 5 | 1 | 20 | 399 | 418 | UGACAGAAGAGAGUGAGCAC | :::::::.:. :::.:: .: | GUGCUCAUUUACUUUUGCUA | Translation | 1 |
| bra-miR156d-5p | BrE2F/DP5 | 5 | 1 | 20 | 399 | 418 | UGACAGAAGAGAGUGAGCAC | :::::::.:. :::.:: .: | GUGCUCAUUUACUUUUGCUA | Translation | 1 |
| bra-miR156e-5p | BrE2F/DP5 | 5 | 1 | 20 | 399 | 418 | UGACAGAAGAGAGUGAGCAC | :::::::.:. :::.:: .: | GUGCUCAUUUACUUUUGCUA | Translation | 1 |
| bra-miR156f-5p | BrE2F/DP5 | 5 | 1 | 20 | 399 | 418 | UGACAGAAGAGAGUGAGCAC | :::::::.:. :::.:: .: | GUGCUCAUUUACUUUUGCUA | Translation | 1 |
| bra-miR156g-5p | BrE2F/DP5 | 5 | 1 | 20 | 399 | 418 | UGACAGAAGAGAGUGAGCAC | :::::::.:. :::.:: .: | GUGCUCAUUUACUUUUGCUA | Translation | 1 |
| bra-miR157a | BrE2F/DP7 | 5 | 1 | 21 | 912 | 931 | UUGACAGAAGAUAGAGAGCAC | ::::::.:.:::: ::::: | UUGCUCUUUGUCUU-UGUCAC | Cleavage | 1 |
| bra-miR162-3p | BrE2F/DP12 | 5 | 1 | 21 | 2172 | 2192 | UCGAUAAACCUCUGCAUCCAG | : :::::::: :: .:::. | UGGCAUGCAGAGUUUGGUCGG | Cleavage | 1 |
| bra-miR164a | BrE2F/DP7 | 5 | 1 | 21 | 1499 | 1519 | UGGAGAAGCAGGGCACGUGCA | ::: : ..:::.::.::.. | AACACAUUUUCUGUUUUUCUG | Cleavage | 1 |
| bra-miR164b-5p | BrE2F/DP7 | 5 | 1 | 21 | 1499 | 1519 | UGGAGAAGCAGGGCACGUGCG | ::: : ..:::.::.::.. | AACACAUUUUCUGUUUUUCUG | Cleavage | 1 |
| bra-miR164c-5p | BrE2F/DP7 | 5 | 1 | 21 | 1499 | 1519 | UGGAGAAGCAGGGCACGUGCG | ::: : ..:::.::.::.. | AACACAUUUUCUGUUUUUCUG | Cleavage | 1 |
| bra-miR164d-5p | BrE2F/DP7 | 5 | 1 | 21 | 1499 | 1519 | UGGAGAAGCAGGGCACGUGCG | ::: : ..:::.::.::.. | AACACAUUUUCUGUUUUUCUG | Cleavage | 1 |
| bra-miR164e-5p | BrE2F/DP7 | 5 | 1 | 22 | 1498 | 1519 | UGGAGAAGCAGGGCACGUGCAA | ::: : ..:::.::.::.. | CAACACAUUUUCUGUUUUUCUG | Cleavage | 1 |
| bra-miR168a-3p | BrE2F/DP10 | 5 | 1 | 21 | 982 | 1002 | CCCGCCUUGUAUCAAGUGAAU | :::::.: ::::.:: :.:: | AUUCAUUAGAUAUAAUGUGGA | Cleavage | 1 |
| bra-miR168a-3p | BrE2F/DP3 | 5 | 1 | 21 | 290 | 310 | CCCGCCUUGUAUCAAGUGAAU | : ::::: :::::::: :: | AAUCACUACAUACAAGGAGGU | Cleavage | 1 |
| bra-miR172c-5p | BrE2F/DP6 | 5 | 1 | 21 | 738 | 759 | GCAUCAUCAU-CAAGAUUCAGA | :::::. :: :::::::::: | AAUGAAUUGUGGAUGAUGAUGC | Translation | 1 |
| bra-miR172c-5p | BrE2F/DP13 | 5 | 1 | 21 | 3948 | 3968 | GCAUCAUCAUCAAGAUUCAGA | :.: :: . ::::::::::: | UUUUAAAUAUGAUGAUGAUGG | Cleavage | 2 |
| bra-miR172d-3p | BrE2F/DP4 | 5 | 1 | 21 | 28 | 48 | GGAAUCUUGAUGAUGCUGCAU | : :::::: :::::::. | CACAACCAUCAUGAAGAUUCU | Cleavage | 1 |
| bra-miR172d-5p | BrE2F/DP12 | 5 | 1 | 21 | 184 | 204 | GCAGCAUCAUUAAGAUUCACA | : .. :: .:::::::::: | GAUCGGCCUCGAUGAUGCUGC | Cleavage | 1 |
| bra-miR1885a | BrE2F/DP8 | 5 | 1 | 22 | 710 | 731 | CAUCAAUGAAAGGUAUGAUUCC | : :::.:::.: :::::: :: | GCAAUUAUAUCAUUCAUUCAUU | Translation | 2 |
| bra-miR391-5p | BrE2F/DP5 | 5 | 1 | 21 | 2234 | 2255 | UUCGC-AGGAGAGAUAGCGCCA | .:::::.:::::: ::::: | UCCUGCUAUUUCUCCUAGCGAA | Cleavage | 1 |
| bra-miR398-3p | BrE2F/DP12 | 5 | 1 | 21 | 1285 | 1305 | UGUGUUCUCAGGUCACCCCUG | :: :::.:::: ::::: | UCAGGAUGAUCUGAUAACACU | Cleavage | 1 |
| bra-miR398-5p | BrE2F/DP12 | 5 | 1 | 21 | 358 | 378 | GGGUCGACAUGAGAACACAUG | :::::: .::.::::: : : | GAUGUGUGUUCGUGUCGUCGC | Cleavage | 1 |
| bra-miR400-5p | BrE2F/DP6 | 5 | 1 | 21 | 270 | 290 | UAUGAGAGUAUUAUAAGUCAC | :.. :.:::::::::: : | UGGGUAUGUAAUACUCUCUUC | Cleavage | 1 |
| bra-miR5654a | BrE2F/DP9 | 5 | 1 | 21 | 838 | 858 | AUAAAUCCCAAGCAUCAUCCA | :::: :::::: :: ::: | UUCAUGACGCUUGGCAUGUAU | Cleavage | 1 |
| bra-miR5654b | BrE2F/DP9 | 5 | 1 | 21 | 838 | 858 | AUAAAUCCCAAGCAUCAUCCA | :::: :::::: :: ::: | UUCAUGACGCUUGGCAUGUAU | Cleavage | 1 |
| bra-miR5713 | BrE2F/DP13 | 5 | 1 | 21 | 763 | 783 | AGGCUUAGAAGAACGUUUGUU | .: .:.. ::::::: ::::: | GAAGAGUCUUCUUCUCAGCCU | Cleavage | 1 |
| bra-miR5713 | BrE2F/DP10 | 5 | 1 | 21 | 764 | 784 | AGGCUUAGAAGAACGUUUGUU | .: .:.. ::::::: ::::: | GAAGAGUCUUCUUCUCAGCCU | Cleavage | 1 |
| bra-miR5713 | BrE2F/DP5 | 5 | 1 | 21 | 757 | 777 | AGGCUUAGAAGAACGUUUGUU | :::. :::::::.:.:: | GCCAAGAAAUCUUCUAGGUCU | Cleavage | 1 |
| bra-miR5716 | BrE2F/DP5 | 5 | 1 | 21 | 602 | 622 | UUGGAUAAUUGAAGAUAUAAA | :::: : ::.:::: ::::: | UUUAAAGGUUUAAUUUUCCAA | Cleavage | 2 |
| bra-miR5716 | BrE2F/DP5 | 5 | 1 | 21 | 3089 | 3109 | UUGGAUAAUUGAAGAUAUAAA | ::: :: :.:::::::..: | CUUAAUUCAUUAAUUAUCUGA | Cleavage | 2 |
| bra-miR5716 | BrE2F/DP12 | 5 | 1 | 21 | 2207 | 2228 | UUGGAUAAUUGAAG-AUAUAAA | :: :: :: :::::::::.: | GGUAGAUACUACAAUUAUCCGA | Cleavage | 1 |
| bra-miR5720 | BrE2F/DP2 | 5 | 1 | 21 | 346 | 366 | UUGUGAUUUGGUUGGAAUAUC | .:.: : ::.:.:::.::: | UGUGUGCGAAUCGAAUUACAC | Cleavage | 1 |
| bra-miR5721 | BrE2F/DP8 | 5 | 1 | 21 | 319 | 339 | AAAAAUGGAGUGAGAAAUGGA | ::.:: .::::::. ::::: | UCUAUAGUUCACUCUUUUUUU | Cleavage | 1 |
| bra-miR5722 | BrE2F/DP12 | 5 | 1 | 22 | 131 | 152 | UGAAAUAGAGUCAUGUGGAACG | :::.:: :::.:: .:::: | UUUUCUACUUGAUUCAGUUUCU | Cleavage | 1 |
| bra-miR5724 | BrE2F/DP5 | 5 | 1 | 21 | 2575 | 2596 | AACCGCCGGUUUGA-UAAUAGC | : :::: :::..:.::.:::: | UCCAUUAAUCAGGCUGGUGGUU | Cleavage | 1 |
| bra-miR824 | BrE2F/DP11 | 5 | 1 | 21 | 517 | 537 | UAGACCAUUUGUGAGAAGGGA | :.: :. ..::::::::.:: | UUCACUUAUGCAAAUGGUUUA | Cleavage | 1 |
| bra-miR9553-5p | BrE2F/DP13 | 5 | 1 | 22 | 1221 | 1241 | UACAAAGCUGAAGCUAAUUAUG | .:::::: ::: ::.:::::. | AGUAAUUAUCUU-AGUUUUGUG | Translation | 1 |
| bra-miR9556-5p | BrE2F/DP2 | 5 | 1 | 21 | 144 | 164 | GUCAAUUGGUGAUAGUAGUUC | :.:. :::: ::::.::: :: | GGAUAACUACCACCGAUUCAC | Cleavage | 1 |
| bra-miR9558-5p | BrE2F/DP4 | 5 | 1 | 21 | 1100 | 1120 | AGAGAUGUCUGGCUUGCAACA | :::::.::.. ::. :::: | CUUUGCAGGCUGAACGACUCU | Cleavage | 1 |
| bra-miR9562-3p | BrE2F/DP8 | 5 | 1 | 21 | 566 | 586 | UUAUUCACAACUGCAUAAUUC | : :::.::..:: :::: :.: | GCAUUGUGUGGUGGUGAUUGA | Cleavage | 1 |
| bra-miR9562-3p | BrE2F/DP13 | 5 | 1 | 21 | 575 | 595 | UUAUUCACAACUGCAUAAUUC | .::: :::.: : ::::::. | UGAUUUUGCGGAUUUGAAUAG | Translation | 1 |
| bra-miR9562-5p | BrE2F/DP9 | 5 | 1 | 21 | 786 | 806 | ACUAUGCAAUUGUGAACAAAC | :: ::::: : ::::::: | AGAUGCUCACAUUCGCAUAGU | Translation | 1 |
| bra-miR9563b-3p | BrE2F/DP6 | 5 | 1 | 22 | 4365 | 4386 | AAAUUAAGAGAUGAAUUCUUAC | ::: :::: :::::: :: | CACUGAACUCAUGUCUUAAGUU | Translation | 1 |
| bra-miR9566-5p | BrE2F/DP13 | 5 | 1 | 22 | 2088 | 2109 | UUGUUGACAAAUACUUAGGCUC | : .. :: :.:::::: :::: | AACUUAAAAUGUUUGUCUACAA | Cleavage | 1 |
| bra-miR9568-3p | BrE2F/DP10 | 5 | 1 | 21 | 733 | 753 | UCAUCGUAAGAGAUCUGCAUU | : : :::.: ::: :.::::: | AUUUCAGGUAUCUGAUGAUGA | Cleavage | 1 |
